# Development and validation of a prospective study to predict the risk of readmission within 365 days of respiratory failure: based on a random survival forest algorithm combined with COX regression modeling

**DOI:** 10.1186/s12890-024-02862-9

**Published:** 2024-02-14

**Authors:** Zhongxiang Liu, Zhixiao Sun, Hang Hu, Yuan Yin, Bingqing Zuo

**Affiliations:** 1 Department of Pulmonary and Critical Care Medicine, The Yancheng Clinical College of Xuzhou Medical University, The First People’s Hospital of Yancheng, Yancheng, 224006 China; 2 Department of Respiratory and Critical Care Medicine, The First Affiliated Hospital of Nanjing Medical Univesity, Nanjing, 210029 China

**Keywords:** Respiratory failure, Nomogram, Readmission, Random survival forest algorithm, COX regression modeling

## Abstract

**Background:**

There is a need to develop and validate a widely applicable nomogram for predicting readmission of respiratory failure patients within 365 days.

**Methods:**

We recruited patients with respiratory failure at the First People’s Hospital of Yancheng and the People’s Hospital of Jiangsu. We used the least absolute shrinkage and selection operator regression to select significant features for multivariate Cox proportional hazard analysis. The Random Survival Forest algorithm was employed to construct a model for the variables that obtained a coefficient of 0 following LASSO regression, and subsequently determine the prediction score. Independent risk factors and the score were used to develop a multivariate COX regression for creating the line graph. We used the Harrell concordance index to quantify the predictive accuracy and the receiver operating characteristic curve to evaluate model performance. Additionally, we used decision curve analysiso assess clinical usefulness.

**Results:**

The LASSO regression and multivariate Cox regression were used to screen hemoglobin, diabetes and pneumonia as risk variables combined with Score to develop a column chart model. The C index is 0.927 in the development queue, 0.924 in the internal validation queue, and 0.922 in the external validation queue. At the same time, the predictive model also showed excellent calibration and higher clinical value.

**Conclusions:**

A nomogram predicting readmission of patients with respiratory failure within 365 days based on three independent risk factors and a jointly developed random survival forest algorithm has been developed and validated. This improves the accuracy of predicting patient readmission and provides practical information for individualized treatment decisions.

## Introduction

Respiratory failure is a common medical emergency that leads to inadequate blood oxygen levels and/or an increase in blood carbon dioxide levels [1]. Patients with respiratory failure often have multiple cardiovascular and pulmonary complications or may be simultaneously suffering from multiple diseases, and therefore often require prolonged hospitalization, ventilatory support, and intensive care unit admission, resulting in high mortality and high readmission rates [2, 3]. To better address these issues, many experts and clinicians have been dedicated to identifying prognostic models for respiratory failure [4, 5]. We chose readmission as our research indicator because it might be expensive for the healthcare system [6], and it represented an economic and clinical burden for patients [7].

A report on specific disease readmission rates suggested that strategies to reduce readmission rates could be successful, such as with heart failure [8, 9]. However, there are few studies on readmission for respiratory failure. Currently, most research focuses on subtypes of respiratory failure or is limited to early readmission studies [10]. Studies also often include patients with respiratory failure who have other co-occurring conditions [11]. Because respiratory failure is a complex chronic disease caused by multiple risk factors, building a predictive model using Cox regression may not be very effective in predicting individual disease risk [12].

Therefore, the establishment of the readmission model for respiratory failure with complex diseases requires not only accurate risk assessment, but also the interpretation of results based on the importance of covariates to evaluate risk factors, with the ultimate goal of developing better diagnostic and treatment strategies [13]. In fact, important covariates may vary due to environmental factors, and from a clinical perspective, all selected covariates are meaningful [14]. In this case, it is necessary to ensure that the designed model has high prediction accuracy without overfitting, and is universally applicable in clinical diagnosis in the real world [15, 16].

In this study, we attempted to use the random forest model in machine learning to solve this problem, which has good processing capabilities for complex high-dimensional data. The purpose of this study was to establish a widely applicable line chart to predict the occurrence of readmission in patients with respiratory failure at 365 days.

## Materials and methods

### Study design

**Fig. 1:**
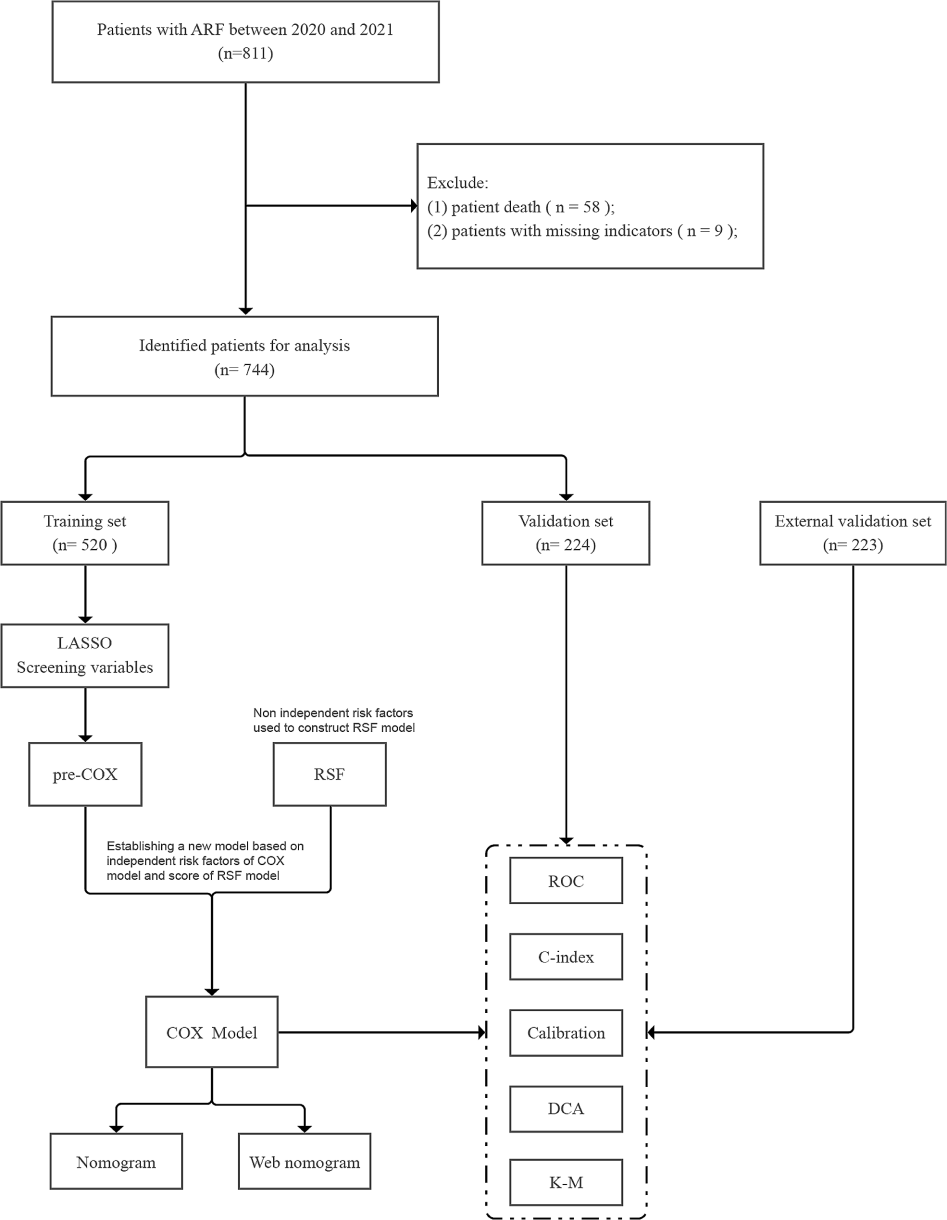
Flow chart of this study

The flowchart of this experiment for a multi-center prospective cohort study was depicted in Fig. 1. The study event was respiratory failure. The start time of the study was when the patient was admitted to the hospital with a diagnosis of respiratory failure based on blood gas analysis.The endpoint of the study were the occurrence of another respiratory failure event and the time point of hospitalization for the event.

First, the samples collected from the First People’s Hospital of Yancheng were divided into a modeling dataset and an internal validation dataset, and then a line chart was proposed for the study work. Second, the model was evaluated using the internal validation dataset. Third, external validation was performed using data provided by the People’s Hospital of Jiangsu Province. The current project followed the principles of the Helsinki Declaration. This study was approved by the ethics committees of the First People’s Hospital of Yancheng (No.2020-K062) and the People’s Hospital of Jiangsu Province (No. 2021-SR-346). In addition, participants from both hospitals provided written informed consent to support clinical research.

### Participants and data collection

We selected 744 patients with respiratory failure who were hospitalized in the First People’s Hospital of Yancheng from October 2020 to September 2021. For external validation, we used a dataset of 223 respiratory failure patients who were hospitalized in Jiangsu Provincial People’s Hospital from October 2021 to December 2021.

The inclusion criteria for research patients were as follows: Arterial oxygen partial pressure (PaO2) was less than 8.0 kPa (60 mmHg) or arterial carbon dioxide partial pressure (PaCO2) was greater than 6.0 kPa (45 mmHg) based on blood gas analysis [17].

Patients with incomplete clinical data, age less than 18 years, death within 24 h, trauma, malignant tumors, malignant hematological diseases, or pregnancy were excluded.

### Follow-up index

The primary indicator for respiratory failure patients was the time from discharge to readmission due to respiratory failure. The information was collected from the two centers mentioned above and followed up for 365 days after discharge. To better track patients, we conducted telephone interviews and used hospital systems to verify the patient’s condition further.

#### Model specification

Firstly, the variables with non-zero coefficients were screened using LASSO regression, followed by multivariate COX regression to identify significant variables. Secondly, the variables excluded by setting their LASSO regression coefficient to 0 and performing multiariable COX regression together were used to construct a new model using the RSF method for prediction. Finally, the meaningful variables identified through COX regression were combined with scores to establish a new multiariable COX regression model.

### Statistical analysis

To ensure comparability between the two groups of patients, 744 respiratory failure patients were randomly divided into two groups, with 70% and 30% of patients in each group, respectively. One group (*n* = 520) was used to develop the Nomogram, while the other group (*n* = 224) was used to verify the predictive ability of the constructed model.

To test the balance between two groups, categorical variables were expressed as frequencies and percentages, and their differences were compared using the Chi-square test. For continuous variables, if they followed a normal distribution, they were expressed as mean ± SD, and their differences were compared using the t-test. If they did not follow a normal distribution, they were expressed as median and quartiles, and their differences were compared using the Mann-Whitney test.

The LASSO regression method was utilized to select significant features from the modeling set for multivariate Cox proportional hazard analysis, screening independent risk factors. The Random Survival Forest (RSF) algorithm was employed to construct a model for the variables exhibiting a coefficient of 0 following LASSO regression, and subsequently compute the prediction Score. The predictive accuracy of the nomogram was quantitatively measured using the Harrell consistency index (C index), which calculated time-related receiver operating characteristic (ROC) curves and areas under the curve to evaluate the model’s performance. The accuracy of the nomogram prediction was evaluated using the calibration curve. Additionally, decision curve analysis (DCA) was used to assess the clinical utility of the nomogram.

Individual risk scores were obtained based on the established nomogram. Risk stratification was determined using the ROC curve to identify the optimal threshold for risk score. The critical value divided patients into high-risk and low-risk groups and provided the best difference for survival analysis between the risk groups. A p-value < 0.05 was considered statistically significant. All statistical analyses were performed using R (version 4.1.3).

## Result

### Baseline characteristics

In this study, we prospectively evaluated a total of 744 patients with respiratory failure who met the inclusion criteria. The evaluation data were summarized and randomly divided into two groups at a ratio of 7:3. Table 1 shows the baseline characteristics of patients in the modeling (*n* = 520) and validation (*n* = 224) cohorts. In the entire cohort, there were 487 males (65.46%) and 257 females (34.54%), with a median age of 74 years. Type 2 respiratory failure patients accounted for 68.15% of the cohort, while type 1 respiratory failure patients accounted for 31.85%. There were 448 (60.22%) smokers. The top three chronic diseases in terms of prevalence were COPD, with 536 (72.04%) patients, hypertension with 289 (38.84%) patients, and pneumonia with 169 (22.72%) patients. Additionally, 186 patients were readmitted, resulting in a readmission rate of 25%. The clinical characteristics between the two groups were well balanced and comparable. The external test set, which met the inclusion criteria, was obtained from the People’s Hospital of Jiangsu Province.

**Table 1:** Demographic and clinical characteristics of patients

Variables	Total (*n* = 744)	Training set (*n* = 520)	Validation set (*n* = 224)	P value	statistic
**status**				0.999	0
0	558 (75)	390 (75)	168 (75)		
1	186 (25)	130 (25)	56 (25)		
**month**	12 (12, 12)	12 (11.975, 12)	12 (12, 12)	0.818	58710.5
**respiratory failure type**				0.883	0.022
1	237 (31.855)	167 (32.115)	70 (31.25)		
2	507 (68.145)	353 (67.885)	154 (68.75)		
**Smoking history**				0.950	0.004
0	296 (39.785)	206 (39.615)	90 (40.179)		
1	448 (60.215)	314 (60.385)	134 (59.821)		
**Gender**				0.983	0
Male	487 (65.457)	341 (65.577)	146 (65.179)		
Female	257 (34.543)	179 (34.423)	78 (34.821)		
**Hypertension**				0.683	0.167
0	455 (61.156)	321 (61.731)	134 (59.821)		
1	289 (38.844)	199 (38.269)	90 (40.179)		
**Diabetes**				0.241	1.378
0	639 (85.887)	441 (84.808)	198 (88.393)		
1	105 (14.113)	79 (15.192)	26 (11.607)		
**Cerebrovascular disease**				0.241	1.378
0	639 (85.887)	441 (84.808)	198 (88.393)		
1	105 (14.113)	79 (15.192)	26 (11.607)		
**Cardiovascular disease**				0.101	2.683
0	592 (79.57)	405 (77.885)	187 (83.482)		
1	152 (20.43)	115 (22.115)	37 (16.518)		
**Chronic bronchitis emphysema**				0.608	0.262
0	208 (27.957)	142 (27.308)	66 (29.464)		
1	536 (72.043)	378 (72.692)	158 (70.536)		
**Asthma**				0.999	0
0	725 (97.446)	507 (97.5)	218 (97.321)		
1	19 (2.554)	13 (2.5)	6 (2.679)		
**Interstitial lung disease**				0.323	0.977
0	702 (94.355)	494 (95)	208 (92.857)		
1	42 (5.645)	26 (5)	16 (7.143)		
**Bronchiectasis**				0.284	1.147
0	657 (88.306)	464 (89.231)	193 (86.161)		
1	87 (11.694)	56 (10.769)	31 (13.839)		
**Pneumonia**				0.378	0.776
0	575 (77.285)	407 (78.269)	168 (75)		
1	169 (22.715)	113 (21.731)	56 (25)		
**length of stay**	10 (7, 14)	10 (7.75, 14)	10 (7, 14)	0.840	58783.5
**height**	165 (160, 170.25)	165 (160, 170.25)	165 (158, 170.25)	0.839	58786.5
**weight**	60 (50, 70)	60 (50, 70)	60 (50, 68.875)	0.593	59675.5
**age**	74 (66, 80)	74 (67, 80)	72.5 (66, 80)	0.097	62699.5
**VTE score**	3 (1, 4)	3 (1, 4)	3 (1, 4)	0.951	58076.5
**White Blood Cells**	7.85 (5.803, 10.803)	7.855 (5.803, 10.825)	7.69 (5.808, 10.498)	0.742	59,126
**hematocrit**	39.2 (34.95, 43.825)	39.05 (34.8, 43.5)	39.3 (35, 44.3)	0.691	57,171
**red blood cell**	4.34 (3.83, 4.78)	4.345 (3.828, 4.77)	4.32 (3.83, 4.802)	0.964	58,362
**Lymphocyte**	0.92 (0.597, 1.322)	0.97 (0.61, 1.35)	0.81 (0.575, 1.223)	0.027	64,193
**hemoglobin**	130 (116, 145)	130 (116, 145)	131 (116, 144)	0.965	58,122
**platelet**	175.5 (130, 225.25)	176.5 (130, 228.5)	172 (128, 223)	0.442	60,310
**neutrophil**	6.125 (4.175, 9.102)	6.07 (4.16, 9.203)	6.33 (4.22, 9.062)	0.818	57,622
**D- dimer**	0.75 (0.39, 1.632)	0.78 (0.41, 1.632)	0.715 (0.338, 1.628)	0.317	60,932
**fibrinogen**	3.63 (2.74, 4.93)	3.6 (2.768, 4.84)	3.66 (2.678, 5.07)	0.800	57557.5
**Glutamyltransferase**	26.7 (17.65, 44.625)	25.05 (17, 43.75)	28 (18.3, 45)	0.199	54,788
**albumin**	35.408 ± 5.122	35.413 ± 5.171	35.397 ± 5.019	0.970	0.038
**glutamic-pyruvic transaminase**	25 (16, 38)	24 (16.375, 37)	25.5 (16, 38)	0.683	57,141
**triglyceride**	0.97 (0.74, 1.36)	1.005 (0.74, 1.4)	0.94 (0.758, 1.232)	0.342	60,797
**creatinine**	65 (53.175, 80.225)	65.5 (53.5, 82.275)	63.95 (52.45, 79.15)	0.630	59536.5
**creatine kinase**	39 (30, 68)	39 (30, 66.55)	41 (30, 70)	0.346	55710.5
**creatine kinase-MB**	10 (8, 15)	10 (8, 14)	10 (8, 17)	0.158	54,465
**alkaline phosphatase**	78.9 (66, 97.325)	78.05 (66, 97)	80.35 (66, 98.25)	0.541	56594.5
**urea**	6.965 (5.12, 8.995)	7.035 (5.135, 9.162)	6.585 (5.092, 8.623)	0.291	61081.5
**globulin**	28.7 (26, 32)	28.85 (26, 32.025)	28.6 (25.85, 31.6)	0.460	60225.5
**lactic dehydrogenase**	353.5 (214.9, 518)	360.5 (217.875, 524.25)	345.5 (207.425, 483)	0.603	59,639
**aspartate aminotransferase**	26 (20, 36)	25.1 (20, 35)	27 (20.325, 37)	0.189	54,709
**total cholesterol**	4 (3.34, 4.74)	4 (3.295, 4.72)	3.985 (3.423, 4.8)	0.717	57266.5
**total bilirubin**	11.96 (8.398, 17.36)	11.9 (8.377, 16.9)	12.345 (8.482, 18.91)	0.447	56,194
**myoglobin**	38.35 (27.1, 65.55)	38.65 (28.15, 66.3)	37.45 (23.8, 64.075)	0.576	59,743
**N-telencephalic natriuretic peptide**	419 (130, 1920)	419.29 (130, 1920)	417.5 (130, 1825)	0.576	59,744
**PO2** ^**2**^	41 (38, 44.25)	41 (38, 44)	40.5 (37.75, 45)	0.891	57870.5
**PCO2**	56 (45, 71)	56 (44.75, 71)	57 (46, 73)	0.304	55,475
**standard bicarbonate**	30.1 (27.3, 33.825)	30 (27.3, 33.9)	30.65 (27.275, 33.7)	0.623	56,916
**methemoglobin**	1.2 (1, 1.3)	1.2 (1, 1.3)	1.1 (1, 1.3)	0.366	60656.5
**reduced hemoglobin**	6.35 (2.7, 10.6)	6.4 (2.7, 10.6)	6 (2.775, 10.35)	0.499	60059.5
**hematokrit**	42 (36, 47)	42 (36, 47)	42 (37, 47)	0.862	57772.5
**base excess**	7 (3.4, 11.625)	6.9 (3.5, 11.8)	7.55 (3.3, 11.4)	0.646	57,005
**lactic acid**	1.5 (1.2, 2)	1.5 (1.2, 2)	1.5 (1.2, 1.9)	0.121	62402.5
**actual bicarbonate**	34 (29.1, 40.325)	34 (29.175, 39.95)	34 (29.1, 40.975)	0.521	56,515
**carboxyhemoglobin**	2.3 (1.8, 2.8)	2.2 (1.8, 2.725)	2.3 (1.9, 2.8)	0.206	54,840
**Blood gas hemoglobin**	13.7 (11.9, 15.125)	13.8 (11.9, 15.2)	13.7 (11.9, 15.1)	0.951	58,073
**oxyhemoglobin saturation**	93.5 (89.075, 97.2)	93.3 (88.975, 97.2)	93.8 (89.2, 97.125)	0.503	56,438
**oxyhemoglobin**	90.3 (86, 93.7)	90.2 (86, 93.625)	90.35 (86.275, 93.725)	0.567	56699.5
**total carbon dioxide**	35.9 (30.5, 42.6)	35.85 (30.5, 42.2)	35.95 (30.575, 43.35)	0.505	56448.5

### Feature selection and nomogram construction

**Fig. 2:**
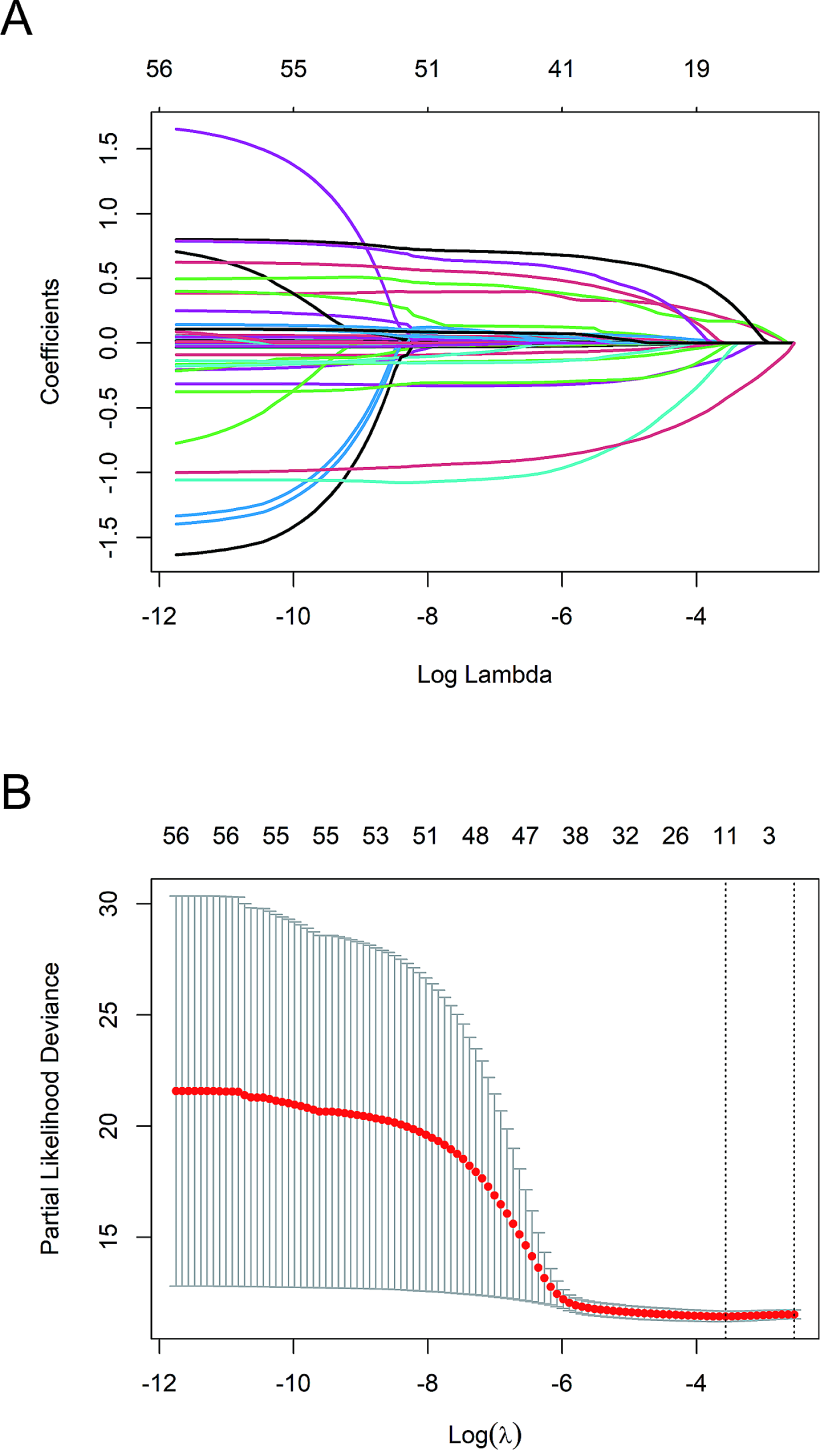
LASSO regression model was used to select feature variables. (**A**) LASSO coefficient curves for the 11 features. (**B**) The adjustment parameter (lambda) in the Lasso regression was selected using 10-fold cross-validation

**Fig. 3:**
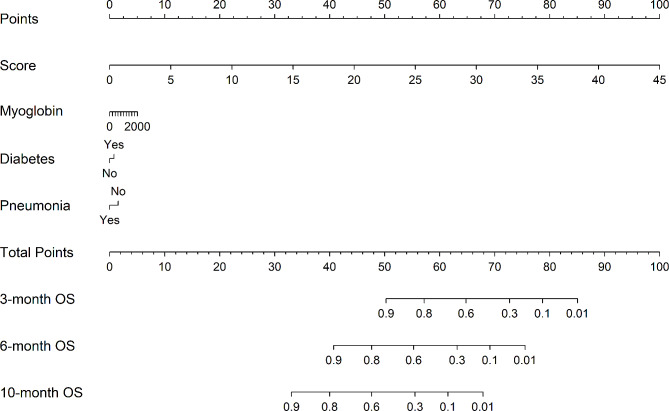
A nomogram predicting readmission risk for respiratory failure

We applied the LASSO regression algorithm to each feature for feature selection in the modeling queue. The biased binomial’s partial likelihood deviance reached its minimum, and the most suitable adjustment parameter λ for LASSO regression was 0.028. Figure 2A displayed the coefficient path generated by the logarithmic λ series values. The LASSO analysis retained 11 variables with non-zero coefficients (Fig. 2B): Glutamyltransferase, triglyceride, total cholesterol, myoglobin, lactic acid, carboxyhemoglobin, respiratory failure type, diabetes, cardiovascular disease, asthma, and pneumonia. Multivariate Cox proportional hazards analysis was performed using eleven variables. Ultimately, three independent risk factors were retained: Myoglobin, Diabetes, and Pneumonia (Table 2). The RSF algorithm was utilized to establish a model for the variables with a coefficient of 0 following LASSO regression and compute the prediction score. Then, the three independent risk factors and prognostic scores were integrated into a multivariate Cox regression model to construct a nomogram based on nomogram, showing the probability of recurrence (Fig. 3). To make the prediction model easy to use, we also developed a web-based format(https://respiratory.shinyapps.io/DynNomapp/).

**Table 2:** Parameters used to develop a predictive model for respiratory failure readmission

Variables	HR	95%CI	P	Chi-square	Estimate	StdErr
**glutamyltransferase**	0.997	0.991–1.002	0.244	-1.165	-0.003	0.003
**triglyceride**	0.778	0.540–1.120	0.177	-1.351	-0.252	0.186
**total cholesterol**	0.900	0.749–1.080	0.257	-1.133	-0.106	0.093
**myoglobin**	0.996	0.993-1.000	0.048	-1.978	-0.004	0.002
**lactic acid**	0.960	0.774–1.190	0.707	-0.375	-0.041	0.110
**carboxyhemoglobin**	1.092	0.902–1.321	0.366	0.904	0.088	0.097
**respiratory failure type**						
1						
2	1.348	0.847–2.144	0.208	1.260	0.298	0.237
**diabetes**						
0						
1	1.974	1.282–3.039	0.002	3.087	0.680	0.220
**cardiovascular disease**						
0						
1	0.698	0.444–1.099	0.121	-1.551	-0.359	0.231
**asthma**						
0						
1	0.318	0.044–2.290	0.255	-1.138	-1.146	1.007
**pneumonia**						
0						
1	0.421	0.235–0.753	0.004	-2.916	-0.865	0.297

### Evaluation and validation of nomogram

A model used to estimate the probability of readmission at 3, 6, and 10 months demonstrated good predictive ability. The C-index was 0.927 (95% CI: 0.910–0.944) in the development cohort, 0.924 (95% CI: 0.901–0.948) in the internal validation cohort, and 0.922 (95% CI: 0.898–0.946) in the external validation cohort.

**Fig. 4:**
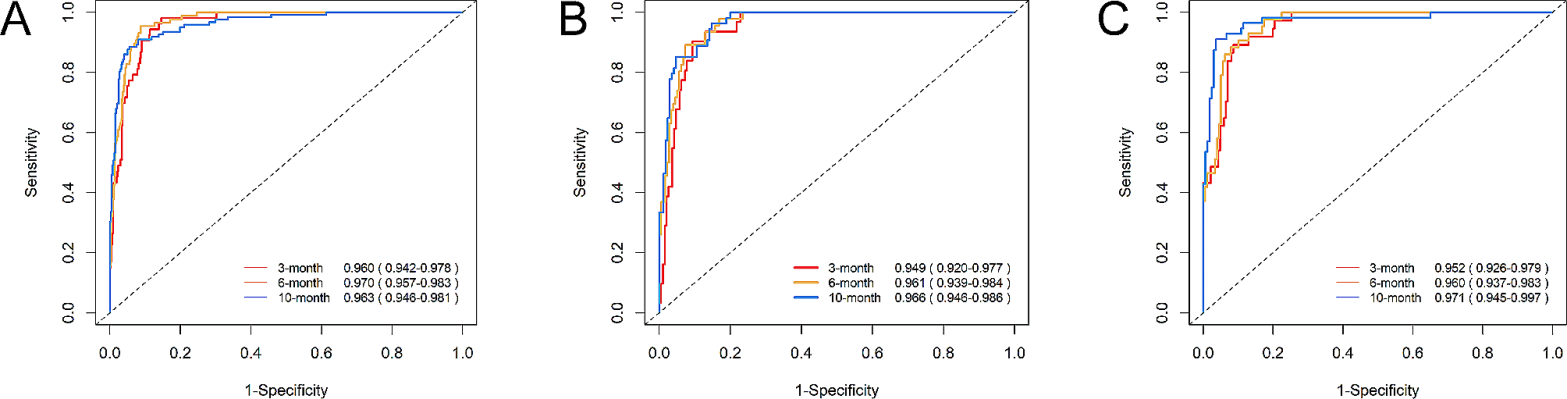
Detection of receiver operating characteristic (ROC) curve. (**A**) the ROC curve of Modeling set. (**B**) the ROC curve of internal validation set. (**C**) the ROC curve of external validation set. The red, yellow, and blue AUC curves show the discrimination of the model at 3, 6, and 10 months. The corresponding 95% confidence interval estimates are highlighted in black text

**Fig. 5:**
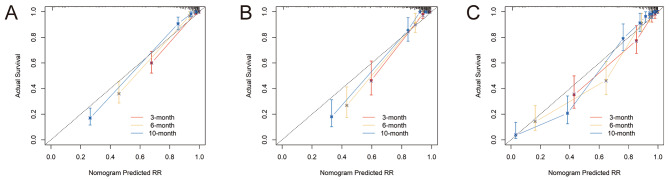
Calibration curve of risk prediction model for respiratory failure readmission. (**A**) the calibration curve of the modeling set. (**B**) the calibration curve of the internal validation set. (**C**) the calibration curve of the external validation set

ROC curve analysis of the line graph in the modeling queue showed excellent classification accuracy, with an AUC of 0.960 (95%CI: 0.942–0.978) at 3 months, 0.970 (95%CI: 0.957–0.983) at 6 months, and 0.963 (95%CI: 0.946–0.981) at 10 months(Fig. 4A). The ROC analysis of the internal validation set confirmed the classification performance, with an AUC of 0.949 (95% CI: 0.920–0.977) at 3 months, 0.961 (95% CI: 0.939–0.984) at 6 months, and 0.966 (95% CI: 0.946–0.986) at 10 months(Fig. 4B). In addition, the ROC analysis of the external test set also verified the classification performance, with an AUC of 0.952 (95% CI: 0.926–0.979) at 3 months, an AUC of 0.960 (95%CI: 0.937–0.983) at 6 months, and an AUC of 0.971 (95%CI: 0.945–0.997) at 10 months(Fig. 4C).The calibration curves for the modeling team, internal validation queue, and external validation queue demonstrate strong consistency between the predicted probability of readmission and the actual occurrence probability at 3, 6, and 10 months(Fig. 5).

### Clinical application of nomogram

**Fig. 6:**
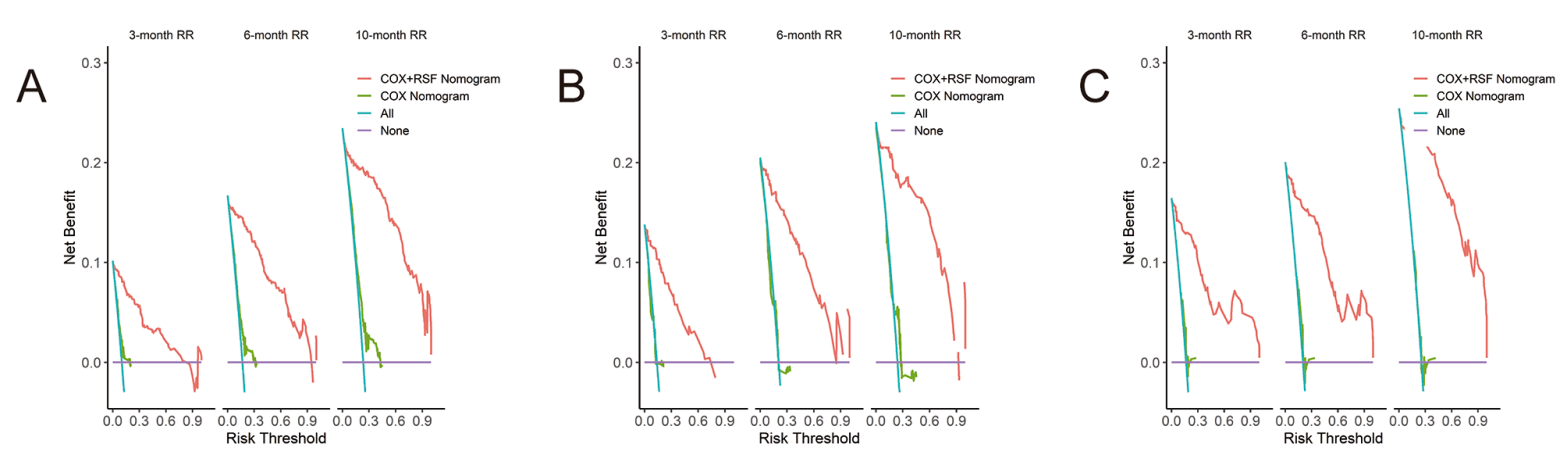
Analysis of decision curve in nomogram. (**A**) the decision curve analysis of nomogram in the modeling set. (**B**) the decision curve analysis of nomogram in the internal validation set. (**C**) the decision curve analysis of nomogram in the external validation set. Solid red lines represent the columns

**Fig. 7:**
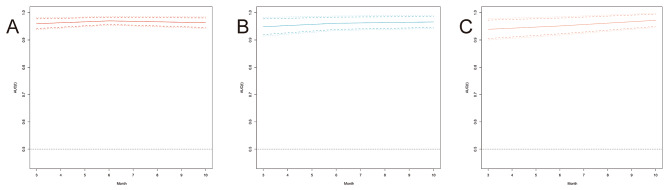
The time-dependent AUC of risk prediction model for respiratory failure readmission. (**A**) the time-dependent AUC of the modeling set. (**B**) the time-dependent AUC of the internal validation set. (**C**) the time-dependent AUC of the external validation set

**Fig. 8:**
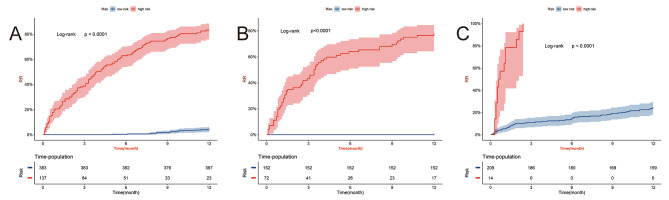
Individual risk scores obtained from the established nomogram. In the modeling set (**A**), internal validation set (**B**) and external validation set (**C**), individual risk scores were obtained according to the established nomogram, and patients were divided into high-risk group and low-risk group according to the critical value to show the best difference in readmission analysis between risk groups

As shown in Fig. 6, the DCA algorithm demonstrated promising clinical value in predicting the probability of readmission at 3, 6, and 10 months. This was evident in the modeling queue, internal validation queue, and external validation queue, where the algorithm outperformed the COX regression model established using three independent risk factors. Specifically, the DCA algorithm utilized a calibration curve to achieve superior performance. The time-dependent AUC revealed consistently higher AUC values for the training set, internal validation set, and external validation set at different time points, indicating a robust and stable discriminative ability of the prediction model across various time intervals (Fig. 7). We used nomogram to calculate the risk value for each patient and then utilized the ROC curve to determine the optimal threshold. Based on this, patients were classified into high-risk (total score ≥ 38.33) and low-risk (total score < 38.33) groups for predicting readmission. The K-M curve illustrated that high-risk patients had a significantly higher readmission rate than low-risk patients across the training, internal validation, and external validation cohorts (Fig. 8).

## Discussion

Our study includes demographic data and clinical information such as body mass index, gender, age, various rating scales, smoking history, and laboratory data (e.g., complete blood count, blood biochemistry, blood gas, etc.). We also consider common and important comorbidities like diabetes, hypertension, cardiovascular disease, and pneumonia. All the features mentioned above are used to predict outcome models.

Improving the predictive form of the RSF model can lead to more accurate individual patient prognoses when establishing a model [18]. The Random Survival Forest algorithm was employed to construct a model for the variables that obtained a coefficient of 0 following LASSO regression, and subsequently determine the prediction score. Additionally, we employed independent risk factors and the score to establish a multivariate COX regression, which was something that traditional scoring systems were unable to achieve.

In our study, we used the Cox model as a baseline predictive model due to its simplicity, allowing for reproducibility and universality [19]. Afterwards, we conducted a random forest survival analysis, in which all predictive factors were included in a single model, using variable importance measures to assess the contribution of each variable to predicting survival [20]. Through the predictive model of readmission risk at 3, 6, and 10 months for patients with respiratory failure, we found that the joint predictive model showed better calibration and discrimination than the single Cox regression model. This model can provide a basis for clinical decision-making. The predictive performance of the model was further validated by employing Time-dependent AUC curves. Our analysis provided insights into predictors of readmission for respiratory failure. We found that patients with acute respiratory failure often returned to the hospital, with pneumonia, diabetes, and myoglobin being identified as the most significant risk factors.

It has been confirmed that pneumonia is the most common cause of readmission for respiratory failure within a year, especially in subgroup analysis of patients undergoing invasive Home mechanical ventilation (HMV) [21]. According to the American Association of Respiratory Care practice guidelines, readmission is usually caused by the worsening of underlying diseases, respiratory tract infections, airway-related side effects, and ventilation failure [22]. In a previous study involving children, 40–70% of discharged patients experienced unplanned readmissions within a short period of approximately 1–3 months after starting HMV, mainly due to pneumonia and respiratory issues [23]. Pneumonia is the main cause of readmission for chronic obstructive pulmonary disease during a one-year period [24]. It can be seen that these reasons for readmission are preventable.

Additionally, patients with acute respiratory failure frequently have elevated blood sugar levels [25]. Diabetes is the most significant risk factor for respiratory failure [26]. Currently, there is no research on the relationship between diabetes and readmission of respiratory failure patients [27, 28]. However, diabetes is a risk factor for readmission of patients with chronic obstructive pulmonary disease [29]. This is mainly because patients with respiratory failure and diabetes are more prone to pulmonary infections, airway mucosal congestion, ciliary dysfunction, and airflow restriction, which can worsen respiratory failure and complicate treatment, necessitating timely intervention with ventilation measures [30].

Similarly, the association between myoglobin and readmission in patients with respiratory failure has not been studied. Myoglobin is an iron- and oxygen-binding protein that is involved in the regulation of the mitochondrial respiratory chain complex IV [31]. Myoglobin plays an important role in oxygen storage in skeletal and cardiac muscles, especially in situations of hypoxemia [32]. Long-term hypoxia can cause non-specific damage to multiple organs. This can lead to increased myoglobin expression, which is related to the degree of hypoxia [33]. Elevated levels of myoglobin in critically ill patients with severe infections, burns, shock, and multiple traumas can predict survival rates and patient prognosis [34]. Studies have shown that myoglobin is a predictive indicator of mortality and risk of deterioration in coronavirus disease 2019 (COVID-19) patients with respiratory failure [35]. Moreover, it has been discovered that elevated levels of myoglobin may be due to other comorbidities, such as chronic obstructive pulmonary disease, cardiovascular disease, and so on [36]. Myoglobin can rapidly be released into the bloodstream as a response to inflammatory stimuli [37]. It is negatively correlated with the percentage of predicted forced expiratory volume in 1 s (FEV1) in chronic obstructive pulmonary disease (COPD) patients [38]. Our results confirm that serum myoglobin levels may be useful in understanding the progression of critical illness, particularly in predicting readmission due to respiratory failure.

Our research has several advantages. Firstly, we provide a simple and feasible tool for identifying patients who are at risk of readmission. By doing so, interventions can be targeted towards reducing readmissions. Secondly, patients with respiratory failure are at high risk for readmission and should be studied as a group to benefit from personalized plans that reduce readmissions.

There are some limitations to this study. First, there may be potential selection bias, such as sample selection bias and patient inclusion bias. Second, larger-scale studies are needed to confirm the predictive ability of the respiratory failure prediction model.

In summary, this study employed a random survival forest algorithm to merge three independent risk factors for respiratory failure. The resulting model provided a simple and user-friendly tool for predicting the probability of readmission within 365 days for patients with respiratory failure. Internal and external validation further demonstrated the broad applicability and reliability of this model in the classification and management of respiratory failure patients, thereby assisting in timely clinical decision-making.

## Data Availability

All data generated or analysed during this study are included in this published article.
